# 7-Ketocholesterol Induces Inflammation and Angiogenesis *In Vivo*: A Novel Rat Model

**DOI:** 10.1371/journal.pone.0056099

**Published:** 2013-02-08

**Authors:** Juan Amaral, Jung Wha Lee, Joshua Chou, Maria M. Campos, Ignacio R. Rodríguez

**Affiliations:** 1 Mechanism of Retinal Diseases Section, Laboratory of Retinal Cell and Molecular Biology, National Eye Institute, National Institutes of Health, Bethesda, Maryland, United States of America; 2 Biological Imaging Core, National Eye Institute, National Institutes of Health, Bethesda, Maryland, United States of America; Washington University School of Medicine, United States of America

## Abstract

Accumulation of 7-Ketocholesterol (7KCh) in lipid deposits has been implicated in a variety of chronic diseases including atherosclerosis, Alzheimer's disease and age-related macular degeneration. 7KCh is known to be pro-inflammatory and cytotoxic to various types of cultured cells but little is known about its effects *in vivo*. In this study we have investigated the effects of 7KCh *in vivo* by implanting biodegradable wafers into the anterior chamber of the rat eye. The wafers were prepared using a mixture of two biodegradable polymers with different amounts of 7KCh. The 7KCh-containing implants induced massive angiogenesis and inflammation. By contrast, no angiogenesis and very little inflammation were observed with cholesterol-containing implants. The neovessel growth was monitored by fluorescein angiography. Neovessels were observed 4 days post implantation and peaked between 7 to 10 days. The angiography and isolectin IB_4_ labeling demonstrated that the neovessels originated from the limbus and grew through the cornea. Immunolabeling with anti-CD68 suggested that the 7KCh-containing implants had extensive macrophage infiltration as well as other cell types. A significant increase in VEGF was also observed in 7KCh-containing implants by fluorescent immunolabeling and by immunoblot of the aqueous humor (AH). Direct measurement of VEGF, IL-1β and GRO/KC demonstrated a marked elevation of these factors in the AH of the 7KCh-implants. In summary this study demonstrates two important things: 1) 7KCh is pro-angiogenic and pro–inflammatory *in vivo* and 2) implants containing 7KCh may be used to create a novel angiogenesis model in rats.

## Introduction

7-Ketocholesterol (7KCh) is a well-studied oxysterol formed by the autooxidation of cholesterol (Ch) and cholesterol esters present in lipoprotein deposits [1]. The oxidation of Ch to 7KCh is known to be caused by catalytic levels of Fe^+2^ and/or Cu^+2^ (known as the Fenton reaction) [1], [2] These transition metals are abundant in the blood and likely able to catalyze the oxidation of lipoprotein deposits in arteries resulting in the formation of atheromatous plaques containing high levels of oxidized LDL and 7KCh [3]. This oxysterol is known to have potent pro-inflammatory and cytotoxic effects on a variety of cultured cells and is suspected of being responsible for much of the toxicity associated with oxidized LDL deposits [1], [3], [4].

In the retina, accumulation of lipids in Bruch's membrane (BrM) and the choriocapillaris as a consequence of aging has been well-documented [5]. While the extent of oxidation of these deposits has not been reported in the human retina, in monkeys, 7KCh has been reported to be localized to lipoprotein deposits in the RPE, BrM and the choriocapillaris [6]. The toxic and inflammatory events that cause 7KCh have been well-studied in vitro [1], [7], [8], but not *in vivo*. *In vitro*, 7KCh has been found to be a potent ER stressor [8], [9], but its precise mechanism of action is unknown and seems to involve a variety of NFκB-related inflammatory pathways [9]–[11].

Various *in vivo* models are available to study ocular angiogenesis. These are mainly the corneal pocket and the laser injury models [12], [13]. The corneal pocket model [12] is usually preferred because of its simplicity and ease of monitoring. However, this model proved to be unreliable in our studies because rats were able to rub off the implants from their corneas, resulting in widely variable results. We have also used the laser injury model and indirectly linked laser-induced CNV to 7KCh-mediated angiogenesis [9]. However, this model is difficult to monitor and quantify since it requires extensive histological manipulations and we were unable to provide a time-course analysis in the same eye [14].

In this study, we are presenting a model for angiogenesis by placing biodegradable implants containing 7KCh in the anterior chamber of the rat eye. We demonstrate that this oxysterol, which is commonly found in atherosclerotic plaques [15]–[17] and in the back of the retina [6], can cause massive inflammation and angiogenesis *in vivo*. Moreover, we also demonstrate that 7KCh can be used to create a very useful model for investigating inflammation-induced angiogenesis.

## Materials and Methods

### Materials

Male Brown Norway rats were purchased from Harlan Laboratories Frederick, MD. Ketamine (100 mg/ml) and Xylazine (20 mg/ml) were purchased from VEDCO Inc., St. Joseph, MO. Pilocarpine Hydrochloride 4% and Proparacaine Hydrochloride 0.5% were purchased from Alcon Pharmaceuticals, Fort Worth, TX. Phenylephrine Hydrochloride 2.5% and Tropicamide Hydrochloride 1% were purchased from Akorn Pharmaceuticals, Lake Forest, IL. 10% Sodium Fluorescein (100 mg/ml) was purchased from Hub Pharmaceuticals, LLC. CA. The nuclear stain 4′,6-diamidino-2-phenylindole (DAPI), isolectin IB_4_ conjugated with Alexa Fluor® 568, and goat anti mouse Alexa Fluor® 488 conjugated secondary antibody were purchased from Molecular Probes, Eugene, OR. Mouse anti rat CD68 antibody (MCA341GA) was purchased from Serotec, Oxford, UK, and mouse anti VEGF antibody (Ab1316) from Abcam, Cambridge, MA. Cytokines were analyzed using the Milliplex MAP Rat Cytokine/Chemokine magnetic bead immunoassay kit from Millipore Corporation, Billerica, MA. The trephines used to prepare the wafers were purchased from Ted Pella; Redding, CA. The straight 30° ophthalmic knives were purchased from Mani Inc., Utsunomiya, Japan.

### Animals use

In the experiments described, 40 rats (150–200 g) were used. The animals were anesthetized intraperitoneally using Ketamine (40–80 mg/Kg) and Xylazine (10–12 mg/Kg). All animal procedures were approved by the National Eye Institute's Animal Care and Use Committee and treated in accordance with the National Institutes of Health guidelines for Animal Care and Use.

### Fluorescein angiography

Neovessel growth was evaluated in 20 animals for up to 21 days by fluorescein angiography [18]. Sedated rats were injected intraperitoneally with approximately 150 µl sodium fluorescein. A fluorescent stereoscopic microscope (NIKON Instruments, Melville, NY) with a camera attachment was used to image the neovessels. Area quantification was performed using the NIKON NIS-Elements analysis software. The area is reported in millimeters squared (mm^2^).

### Histologic evaluation

Eyes from rats containing 20% 7KCh and 20% Ch implants (6 rats each) were removed and fixed in 4% paraformaldehyde for 1 h. The tissues were washed and cryoprotected with a 20% sucrose solution, then embedded in optical cutting temperature (OCT, Sakura Fine Tech. Torrance, CA) using cold acetone. Processed eyes were kept at −80°C until used. Cryosections for immunohistochemistry were 10 µm thick. Sections were washed with cold ICC buffer (0.5% BSA, 0.2% Tween 20, 0.05% sodium azide) in PBS. A 1∶1000 dilution of a 1 mg/ml solution of DAPI, and a 1∶100 dilution of a 1 mg/ml solution of isolectin IB_4_ were prepared in ICC buffer and centrifuged for 1 minute at 5,000 rpm. The sections were incubated for 30 minutes. Macrophages were detected using an anti-CD68 antibody at 1∶100 dilution. VEGF was detected using an anti-VEGF antibody, also at 1∶100. Sections were incubated overnight and the immunoreactivity developed with an Alexa Fluor® 488 conjugated secondary antibodies. Sections were mounted with Fluoro-gel in Tris Buffer (EMS, Hartsfield PA), covered, and sealed.

### Anterior Chamber Paracentesis

Rats were sedated under general (ketamine/xylazine) and local anesthesia (proparacaine), and their pupils were dilated (tropicamide and phenylephrine). A 33G Flexifil beveled needle (World Precision Instruments, Sarasota FL) attached to a 100 µl Hamilton syringe was inserted through the cornea into the anterior chamber and 20 µl of aqueous humor (AH) was withdrawn from each eye. The AH samples from two eyes (∼40 µl) were pooled and collected at 4 and 7 days after implantation. The samples were placed in micro tube and stored at −80°C until ready for analysis. Measurements at day 2 were attempted but the corneal wound was not fully healed. Consequently, AH samples were contaminated with blood, making cytokines measurements unreliable.

### Immunoblot analysis of VEGF

AH samples (5 µl/lane) were separated in 4–12% NuPAGE Novex Bis-Tris Gels running in 1X NuPAGE MOPS SDS Running Buffer at room temperature for 50 min at 200 V (Invitrogen Corp, Carlsbad, CA). The proteins were transferred using the Invitrogen iBlot transfer system. The blot was stained in Ponceaus S solution (Sigma-Aldrich, St. Louis, MO) for rapid detection of protein bands on nitrocellulose membrane. The blot was imaged for future reference and the ponceau red removed with subsequent water washes. The blot was then blocked in 1X TBS, pH 7.4, 5% nonfat milk, and 0.05% Tween-20, for 1 h at room temperature then incubated with mouse anti-VEGF (ab1316, Abcam, Cambridge, MA) at 1∶1,000 dilutions overnight at 4°C. The blot was developed using anti–mouse IgG peroxidase–conjugated secondary antibody (Cell Signal Technology, Inc., Danvers, MA) at a dilution of 1∶1,000 or for 1 h, followed by incubation with the chemiluminescent substrate (SuperSignal West Pico; Thermo Fisher Scientific, Rockford, IL). The total protein content was measured using the BCA method (Thermo Fisher Scientific, Rockford, IL).

### Cytokine assay in AH

The levels of IL-1β, IL-6, MIP-1α, TNF- α, GRO-KC and VEGF were measured in AH samples using a magnetic bead immunoassay kit following the manufacturer's protocol (Luminex Corp, Austin, TX). Briefly, 25 µl of diluted AH sample (5 µl of AH) was added to the pre-wet plate containing 25 µl of assay buffer. To each well, 25 µl of multiple premixed microbeads were added and the plate was sealed with a plate sealer and incubated with agitation on a plate shaker for 18 h at 4°C. The wells were washed twice with 200 µl Milliplex wash buffer. The beads were shaken with 25 µl of the detection antibody mix for 2 h at room temperature; each antibody is specific to a single cytokine/chemokine. The beads were shaken with 25 µl of the streptavidin–phycoerythrin solution for 30 min at room temperature and washed twice again. The beads were suspended in each well with 125 µl of the Milliplex Sheath fluid in a shaker for 5 min. The plate was read on a MAGPIX Luminex xMAP instrument (Luminex Corp, Austin, TX) and analyzed with MILLIPLEX Analysis software version 3 (EMD Millipore Corporation, Billerica, MA).

The concentration of each cytokine was determined using the array reader. A parallel standard curve was constructed for each cytokine. The cytokine levels were normalized to 1 ml of AH.

### Statistical analysis

Statistical analysis was assessed using one-way ANOVA with a 95% confidence interval. Bonferroni posttest was used for analysis of the different conditions (GraphPad Prism 5). A P value≤0.05 was considered statistically significant.

## Results

### Preparation and implantation of 7KCh-containing wafers

7-KCh has been previously reported to be a very potent inducer of VEGF *in vitro* [10], [11], [15] but its angiogenic potential *in vivo* has not been previously demonstrated. In order to investigate its pro-angiogenic properties, 7KCh-containing implants were placed in the anterior chamber of the rat eyes and the angiogenic response monitored by fluorescein angiography [18].

Wafers were prepared by mixing poly (2-hydroxyethylmethacrylate) and polyethylene glycol (mw 20,000) in equal portions and dissolved in ethanol by heating to 45°C in a water bath sonicator at a concentration of 100 mg/ml (50 mg/ml each). A 10 mg/ml solution of 7KCh was prepared separately also in ethanol. The two solutions were mixed to make final concentrations of 5, 10, 15 and 20% 7KCh (w/w). During the removal of the ethanol, the solids precipitated unevenly making it difficult to know if the three white compounds were fully mixed. To ensure homogeneity the mixture was mixed well with a spatula while in semi-solid state and a drop of India ink added to visualize the homogeneity before completely removing the remaining ethanol under vacuum. An identical mixture containing 20% Ch was also made for use as control. The powdered mixture (50 mg) was placed into a 20 mm die and subjected to 25 metric tons of pressure using a hydraulic press (SPECAC Ltd, UK). The thickness of the wafer was approximately 0.2 mm. The implants were prepared by punching 0.5 mm disc from the main wafer using a trephine.

**Figure 1:**
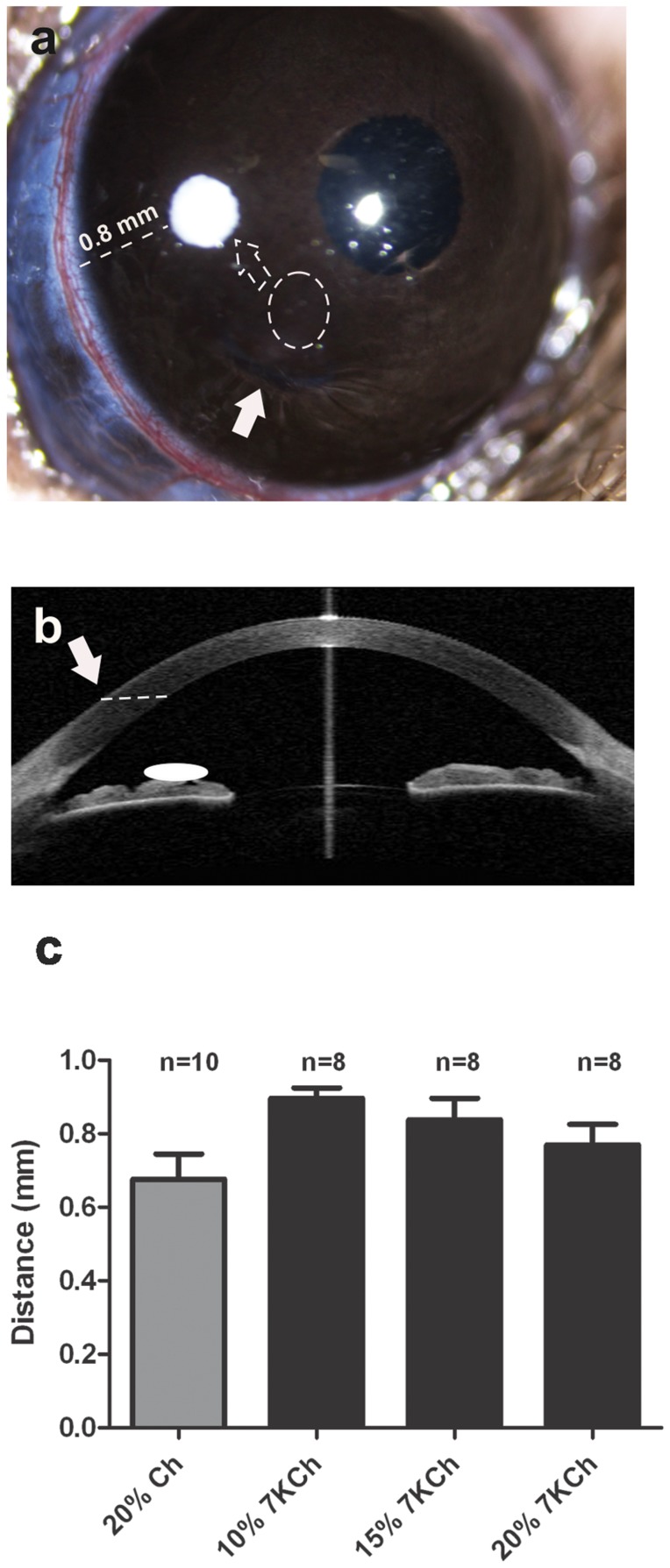
Wafer implantation into the rat anterior chamber. A. A full thickness corneal incision was made parallel to the plane of the iris (arrows). Wafers were introduced to the anterior chamber using toothless forceps (dotted circle), and displaced to its temporal position (dotted arrow) in the anterior chamber. B. Side-view schematic showing the approximate location of the implant. C. The final position of all implants was an average of 0.8 mm from the limbus (dotted line). Ch = cholesterol, 7KCh = 7-ketocholesterol, n = number of wafers implants.

The implantation of the 7KCh-containing wafers was performed by making a full thickness mid-corneal incision using a 30° ophthalmic knife (Fig. 1). Using toothless forceps the wafer implants were introduced into the anterior chamber and displaced toward its final location in the temporal area approximately 0.8 mm from the limbus (Fig. 1a). A schematic of the side view is shown in Fig. 1b. The average distance from the limbus for the various implants is shown in Fig. 1c.

### Angiogenic response to 7KCh-containing implants

**Figure 2:**
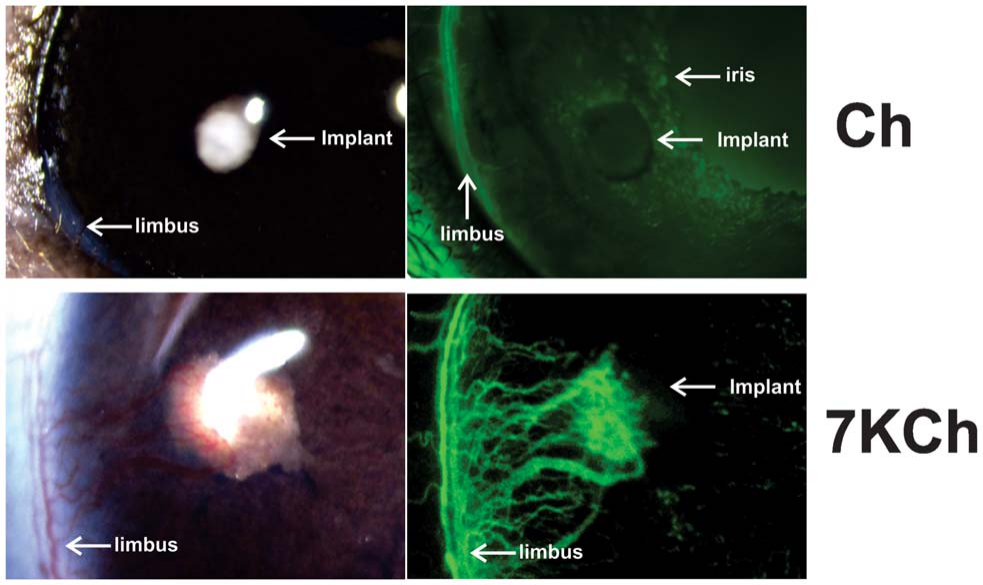
Angiogenesis in Ch versus 7KCh implants. Ch (20% w/w) and 7KCh (10% w/w) implants were placed in the anterior chamber of the rat eye as described in Fig. 1. Both implants were imaged 7 days post implantation with and without fluorescein angiography. Arrows mark the locations of the iris, limbus and implants.

The 7KCh (10%, w/w) and Ch (20%, w/w) implants were inserted into the anterior chamber of the rat eye as described above (Fig. 1). The implants were imaged 7 days post implantation (Fig. 2). As clearly demonstrated, 7KCh displayed very robust angiogenesis, sharply contrasting with the Ch implants, which had essentially no angiogenesis (Fig. 2). The images demonstrate two things: 1) that the majority of the neovessels originate from the limbus and not the iris and 2) the 7KCh implant grows in size and is at least twice the diameter of the Ch implant.

### Effects of 7KCh concentrations on angiogenesis

**Figure 3:**
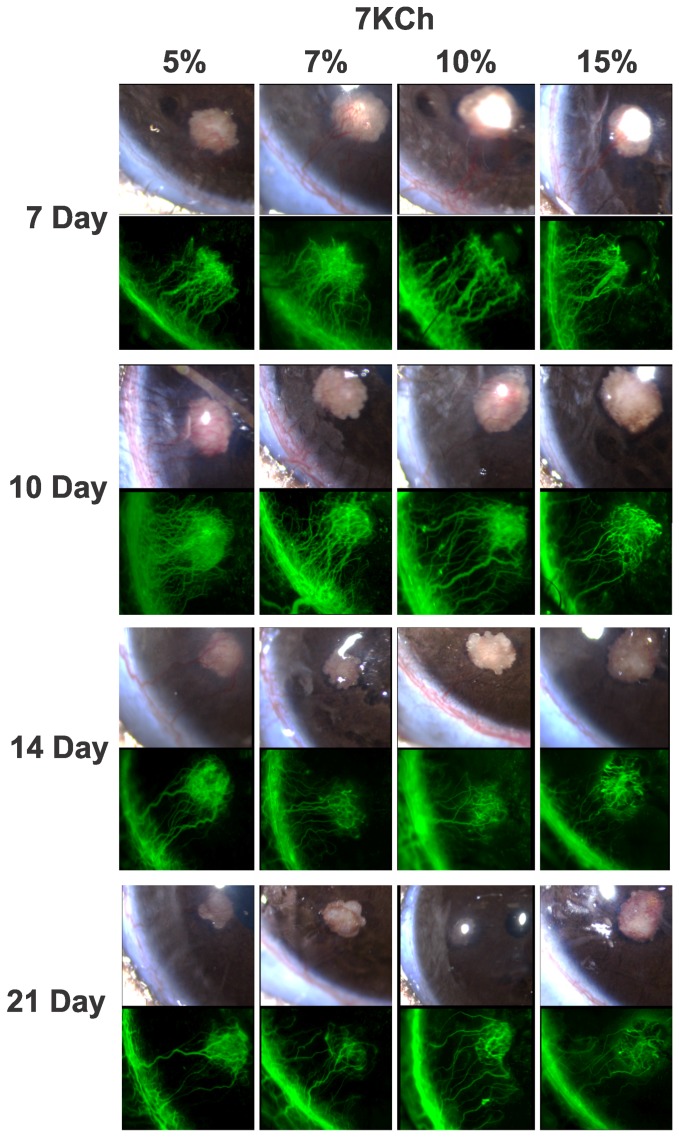
Effects of time and 7KCh concentrations on angiogenesis. Wafers containing 5, 7, 10 and 15% 7KCh (w/w) were implanted into rats anterior chambers as described in Fig. 1 and representative images are shown at 7, 10, 14 and 21 days. The vessel volume for each panel is shown in Fig. 4b, in mm^2^.

We tested various 7KCh concentrations to determine how they affected the degree and duration of the angiogenesis. The results from 5, 7, 10 and 15% 7KCh implants are shown in Fig. 3. 7KCh concentrations below 5% did not consistently induce angiogenesis (data not shown). For example, implants containing 3% 7KCh were only 50% effective at generating angiogenesis (data not shown). Representative images of the inflammatory response to wafers containing 5, 7, 10 and 15% 7KCh are shown in Fig. 3 from day 7 to day 21. There seems to be little difference in the extent or the duration of the angiogenesis in 7KCh concentrations between 5–15%. In all concentrations, angiogenesis peaked between 7 to 10 days and significantly waned by day 21. Similar results were obtained with 20% 7KCh implants (data not shown). Implants containing 5% 7KCh was >80% effective at generating angiogenesis (data not shown). These results indicate that 5% 7KCh is the minimal dose needed to achieve consistent angiogenesis and larger doses do not significantly prolong or increase the levels of angiogenesis.

### Measurement of the angiogenic response

**Figure 4:**
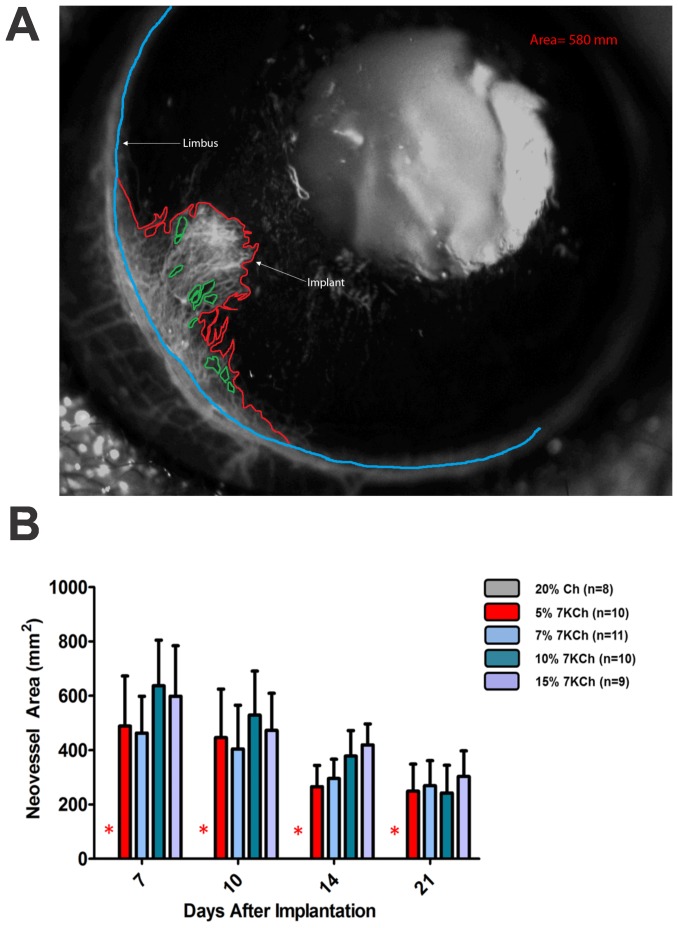
Technique used to quantify neovessel formation. Vessels were imaged using a fluorescent dissecting microscope immediately after IP injection of fluorescein as described in Methods. A. Neovessel area was quantified using Nikon NIS elements hands free tool by delineating the base of neovessels (blue line), the border of the neovessels (red line) and subtracting areas with no vessels (green line). B. Graph showing neovessel area from the experiments described in Fig. 3 in mm^2^.

To quantify the neovessels fluorescein angiograms were performed and images taken at different time points. The NIS-elements software (Nikon, Inc.) was used to measure the areas covered by the neovessels (Fig. 4). The base of the neovessels area was defined as the continuation of the anterior border of the limbus which can be easily identified by fluorescein angiography (blue line). Once the base was defined, the border of the neovessels was outlined (red line) and the zones without vessels (green lines) subtracted (Fig. 4a). The net neovessel areas for the experiments depicted in Fig. 3 are shown in Fig. 4b. Vessel area is reported in mm^2^.

### Immunohistochemical localization of corneal neovessels

To better characterize the evolution of the corneal neovascularization, cryosections of the 7KCh and Ch implants were made 2, 4, 7, and14 days after wafer implantation. To localize the corneal neovessels the cryosections were stained with Alexa Fluor® 568 isolectin IB_4_ which is an endothelial cell marker. The nuclei were stained with DAPI.

**Figure 5:**
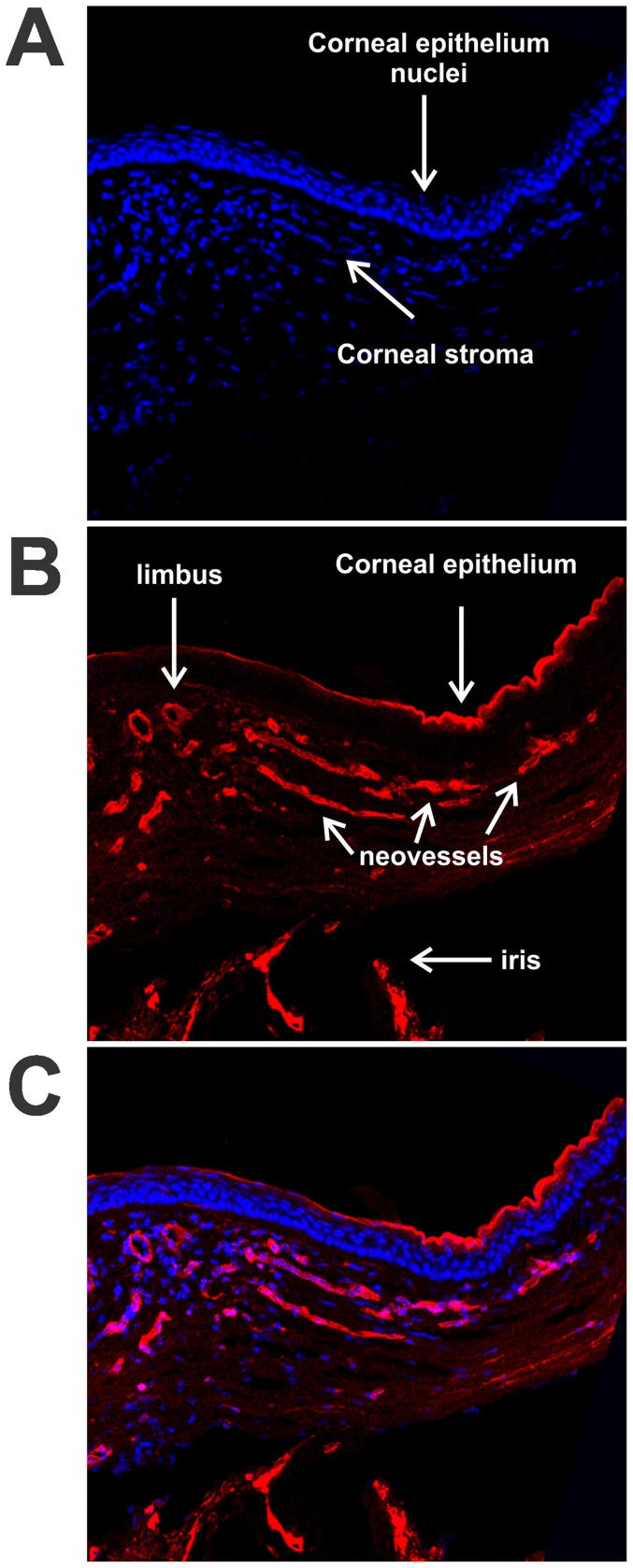
Localization of corneal neovessels with isolectin IB_4_ . Cryosections of temporal corneas were prepared 14 days after implantation with a 20% 7KCh wafer. The sections were labeled with AlexaFluor 568 Isolectin IB_4_ (red) and the nuclei stained with DAPI (blue). A. DAPI stained section demonstrating the location of the corneal stroma and epithelium. B. Isolectin IB_4_ labeling of neovessels, corneal epithelium and iris. C. Combined image. Arrows mark the different structures.

Bowman's membrane is not well defined in rodents and thus could not be used to identify the anterior limbal border histologically. Therefore, the point where the corneal epithelium changes to a transitional epithelium was used as the anterior limbal landmark (Fig. 5a). In Fig. 5 we demonstrate a corneal cross section of a 20% 7KCh implant 14 days after implantation. The corneal epithelium and stroma are marked in Fig. 5a using the DAPI nuclear stain (blue). The original limbus vessels and the neovessels are marked by isolectin IB_4_ (Fig. 5b). The composite of both colors is shown in Fig. 5c. Please note that isolectin IB_4_ also labels the corneal epithelium and the iris. This figure clearly demonstrates that the neovessels grow through the corneal stroma.

### Histological examination of implants

**Figure 6:**
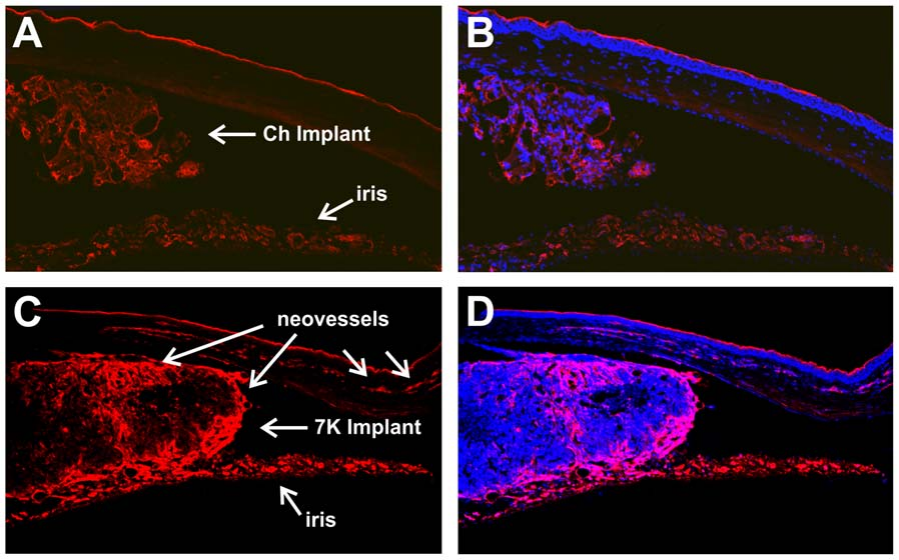
Histological comparison of Ch and 7KCh implants using isolectin IB_4_. Rat eyes were implanted with 20% Ch and 7KCh wafers and cryosections prepared 14 days post implantation. A. Ch implant labeled with isolectin IB_4_. B. Ch implant dual labeled with DAPI. C. 7KCh implant labeled with isolectin IB_4_. D. 7KCh implant dual labeled with DAPI.

Implants containing 20% sterols were removed 14 days after implantation and examined histologically (Fig. 6). The cross sections were labeled with isolectin IB_4_ (red) and nuclei stained with DAPI (blue). Comparing the Ch and the 7KCh-containing implants, it can be clearly seen that the Ch implant is significantly smaller, contains fewer cells and no neovessels can be seen in the cornea (Fig. 6 a, b). By contrast, the 7KCh-containing implant is more than twice as large and is infiltrated with many more cells and neovessels (Fig. 6 c, d). The Ch implant is detached from the iris while the 7KCh implant seems to be attached to both the iris and cornea. Since the iris stains with the isolectin IB_4_ it is difficult to know if any neovessels originate from the iris. However, the fluorescein angiograms shown in Figs. 2 and 3 suggest that the iris is not providing a significant number of neovessels.

### Localization of CD68 positive cells (macrophages) and VEGF in the implants

**Figure 7:**
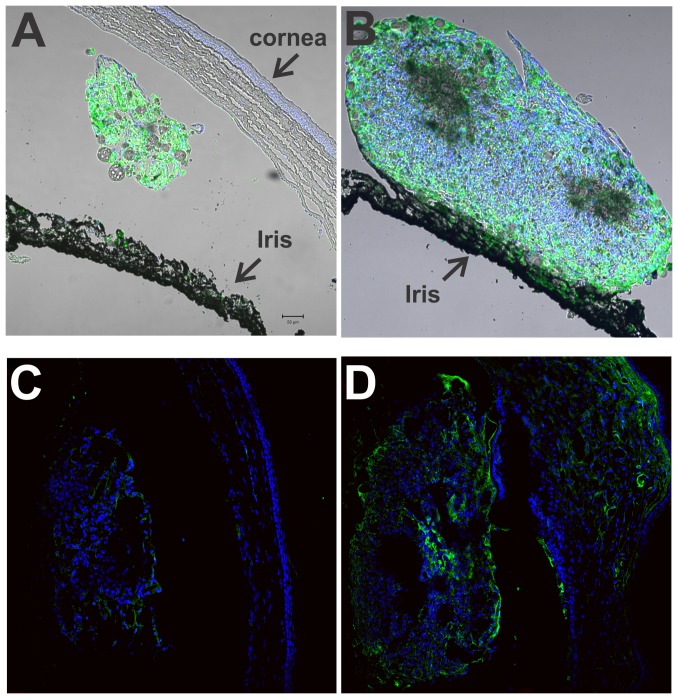
Localization of CD68 positive cells and VEGF in Ch and 7KCh implants. Rat eyes were implanted with 20% Ch and 7KCh wafers and cryosections prepared 14 days post implantation. The sections were labeled with AlexaFluor 488 (green) for anti-CD68 or anti-VEGF and the nuclei stained with DAPI (blue). A. Ch implant, anti-CD68. B. 7KCh implant, anti-CD68. C. Ch implant, anti-VEGF. D. 7KCh implant, anti-VEGF.

Macrophages are known to respond to oxidized LDL and 7KCh [15]–[17], [19]. Thus, to further evaluate the implants, cross sections were labeled with anti-CD68, a rat macrophage marker (Fig. 7), and the nuclei again stained with DAPI. Comparing the two implants, the Ch implant (Fig. 7a) is considerably smaller than the 7KCh implant (Fig. 7b). The Ch implant seems to be freely floating and unattached from the iris or cornea. By contrast, the 7KCh implant is much larger and in this section is seen clearly attached to the iris (Fig. 7b). Both implants contain CD68 positive cells. In the 7KCh implants, CD68 positive cells are in the periphery and non-CD68 expressing cells in the center of the implant.

7-KCh is known to induce VEGF mRNA and protein levels in cultured cells [10], [11] but little is known about its induction *in vivo*. 7KCh and Ch implants were analyzed by immunofluorescence using an anti-VEGF antibody (green). The cryosections were also stained with DAPI to highlight the cell nuclei. Anti-VEGF staining can be observed in the 7KCh implant and in the corneal neovessles (Fig. 7 d). The Ch implants showed minimal or no staining for VEGF (Fig. 7c).

### Measurement of VEGF in AH

**Figure 8:**
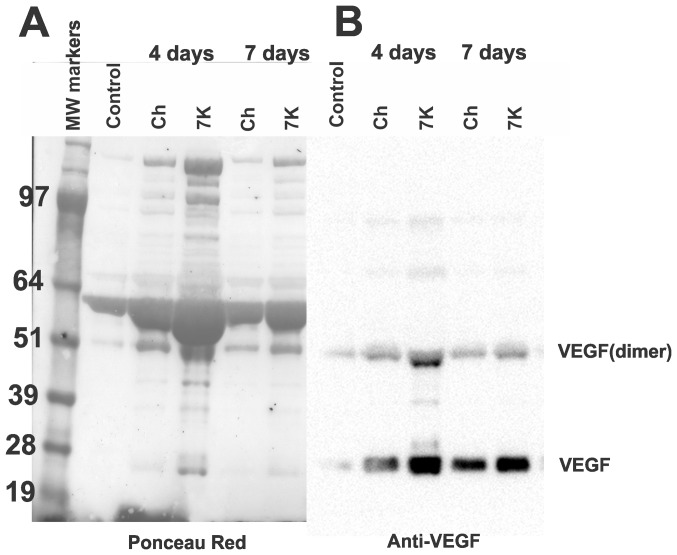
SDS-PAGE and immunoblotting of AH from Ch and 7KCh implants. AH (5 µl) from control (un-implanted eye), Ch and 7KCh implanted eyes were separated by SDS-PAGE and blotted. A. Ponceau S red stained blot demonstrating the difference in protein content between samples. B. Anti-VEGF labeling of blot.

VEGF is a secreted peptide, thus, the staining observed in the fixed implants and corneal neovessels is likely intracellular VEGF or VEGF bound to its receptors. The majority of the secreted VEGF is likely to be present in the AH. Hence, we extracted AH from eyes that were untreated (control) and implanted with 20% Ch and 20% 7KCh implants, then performed immunoblot analyses to detect VEGF (Fig. 8). The AH of implanted animals demonstrated a marked increase in protein concentration (Table 1). At 4 days after implantation, the protein in the AH of the Ch- and 7K-implanted anterior chambers increased 3.4-fold and 6.6-fold over the untreated control, respectively (Table 1). By day 7 the levels were lower but still elevated over control. This was again demonstrated when identical volumes of AH samples (5 µl) were separated by SDS-PAGE. The blot was stained with ponceau red (Fig. 8a) before it was developed with anti-VEGF (Fig. 8b). The VEGF levels are seen to be markedly elevated by day 4 and remain elevated by day 7 (Fig. 8 b). The AH from the 7KCh-implant contains considerably higher amounts of both monomer and dimer forms of VEGF (Fig. 8b). Interestingly, both forms of VEGF seem to be constitutively expressed in the AH (Fig. 8b).

**Table 1:** Protein concentration of aqueous humor.

	Protein
Control	1.42 mg/ml
Day 4 implants	
Ch	4.84 mg/ml
7KCh	9.41 mg/ml
Day 7 implants	
Ch	2.23 mg/ml
7KCh	3.81 mg/ml

### Quantification of VEGF and other cytokines in AH

**Figure 9:**
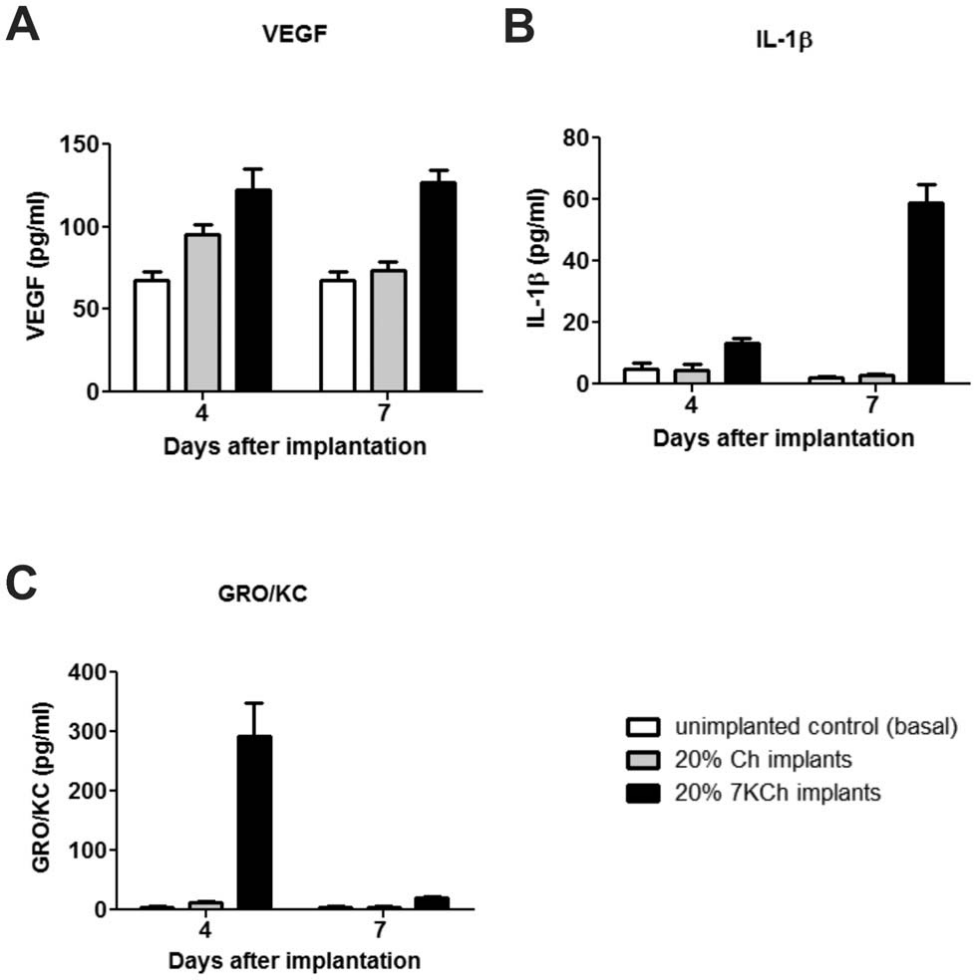
Quantification of VEGF, IL-1β and GRO/KC in AH 4 and 7 days after implantation. AH from eyes of un-implanted (control) and implanted with 20% Ch and 7KCh wafers were analyzed for cytokine levels 4 and 7 days post implantation, as described above. A. VEGF. B. IL-1β. C. GRO/KC.

7-KCh is known to induce various cytokines and chemokines in vitro [10], [11], [15] but these have not been measured *in vivo*. In order to quantify the induction of cytokines and chemokines involved in angiogenesis, 20% wafers of Ch and 7KCh were implanted in a total of four rats, two rats for each condition. Within each condition, the AH was removed from one rat at day 4 and then from the other rat at day 7. The levels of VEGF, MIP-1α, IL-1β, IL-6, TNF- α and GRO/KC were quantified as described in Material and Methods. AH from Ch implants and from un-implanted animals were used as controls. The basal levels of MIP-1α, IL-6 and TNF- α were essentially nil and there was no measurable induction of these cytokines in the AH in any of the implants (data not shown). VEGF levels were elevated in the AH of 7KCh implants at both time points (Fig. 9a). IL-1β increased approximately 3-fold over controls by day 4. By day 7 the IL-1β levels had increased by approximately 21-fold (Fig. 9b). The response by GRO/KC was more marked increasing by approximately 32-fold over controls by day 4 (Fig. 9c) and then dropping to nearly basal levels by day 7.

## Discussion

In this study we have developed a novel angiogenesis model by implanting 7KCh-containing wafers in the anterior chamber of the rat eye. This model has several advantages over other angiogenesis models which make it particularly useful for drug testing. It is relatively inexpensive, technically easy to generate and doesn't distort the cornea. This facilitates imaging and quantification of the neovessels (Figs. 1, 2, 4). The main difference between the corneal pocket and the anterior chamber models is reliability. We could obtain 100% angiogenesis in all anterior chamber implanted eyes using 7KCh concentrations at or above 5% while implants placed in corneal pockets only generated angiogenesis in 50% of the animals regardless of 7KCh concentration. We believe this is due to the ability of the rats to rub the implant off their corneas. In addition, corneal distortion in the corneal pocket model makes it difficult to generate the high quality images needed for accurate neovessel area quantification. This study also demonstrates that 7KCh is pro-inflammatory and pro-angiogenic *in vivo*. The cytotoxic and inflammatory effects of 7KCh have been extensively studied and published *in vitro* [1], [3]–[11], [15] but to our knowledge this is the first report directly implicating 7KCh in angiogenesis *in vivo*.

7-KCh was able to induce angiogenesis as early as 4 days post implantation. As mentioned above, at concentrations of 5% or greater 7KCh generated neovessels in 100% of all of the implanted rats (Fig. 3). The neovessel area peaked at 7 to 10 days and was significantly reduced by 21 days (Fig. 4b). Histological sections through the implant area indicate that the blood vessels originate at the limbus and grow through the cornea (Figs. 5 and 6). No vessels were seen in the Ch control (Fig. 6a). The 7KCh implants attract large numbers of cells and by day 14 are several times larger than the Ch implants (Fig. 7a,b). Both implants contain CD68 positive cells (presumed macrophages) but in 7KCh implants these cells concentrate around the periphery (Fig. 7b). The 7KCh implants were firmly attached to the cornea and iris and have numerous blood vessels growing through and around them (Figs. 6 and 7) while roughly doubling in diameter. By contrast, the Ch implants have much fewer cells and remain unattached to the cornea or iris (Figs. 6a and 7a). Unlike the 7KCh implant, the Ch implants only seem to have CD68 positive cells (macrophages) and are reduced to roughly half of their original size (Fig. 7a). The appearance of CD68 positive cells in the Ch implant seems to be the result of trauma to the iris epithelial cells during implantation. Some pigmented cells can be seen attached to the Ch implant (Fig. 7a). The introduction of the wafer implant into the anterior chamber and its subsequent temporal displacement seems to scrape the iris and detach some cells that subsequently attract macrophages. Nevertheless, the immune response caused by this trauma is clearly minor when compared to the response generated by the 7KCh implants.

The role of VEGF in angiogenesis has been well established [19], as well the use of anti-VEGF therapies for the treatment of ocular angiogenesis [20]. Histological sections of the implants demonstrated anti- VEGF immunoreactivity in the 7KCh (Fig. 7d) but not in Ch-implants (Fig. 7c). This is consistent with the VEGF induction previously demonstrated *in vitro* [10], [11]. However, since VEGF is a secreted protein we examined the AH of the implanted rats. The first thing we noticed was the markedly elevated protein concentration in the AH of the implanted eyes (Table 1, Fig. 8a). In order to get a better understanding of the angiogenic response, the chemokines MIP-1α and GRO/KC and the cytokines VEGF, IL-1β, IL-6 and TNF- α were measured at 4 and 7 days post implantation. These molecules are known to be involved in inflammatory responses and angiogenesis. The results clearly show that the implantation process itself can raise the levels of these molecules as seen with the Ch implants (Fig. 9). However, the 7KCh-containing implants cause a significantly more robust and prolonged response, which is consistent with the neovessels formation and the histological data. MIP-1α, IL-6 and TNF- α were not detectable in AH (data not shown), but VEGF, IL-1β and GRO/KC were markedly induced in the 7KCh-implant (Fig. 9). VEGF essentially doubled by day 4 and this level was sustained through day 7 (Fig. 9a). IL-1β levels tripled over controls by day 4 and further increased to 21-fold by day 7 (Fig. 9b). The GRO/KC increased approximately 32-fold over controls by day 4 but the levels significantly dropped by day 7 to only 2-fold (Fig. 9c). VEGF has a relatively minor induction (2-fold) when compared to IL-1β and GRO/KC which warrants further investigation. Both of these cytokines are known to be synthesized by macrophages and are involved in angiogenesis [21], [22]. However, the IL-1β was considerably greater at day 7 than at day 4. Since neovessels are already present by day 4 this suggests IL-1β may not be playing a primary role in the 7KCh-mediated angiogenesis. The fact that VEGF is constitutively expressed in the AH also suggests that it may be playing a “housekeeping” function and its role in inflammatory angiogenesis is dependent on the induction of other factors, like GRO/KC. GRO/KC responds vigorously early-on (day 4) but is nearly back to basal levels by day 7 when the neovessel growth is reaching its peak. This suggests that GRO/KC may be playing a more direct role in the 7KCh-mediated angiogenesis. On a side note, the retinas of the 7KCh implanted rats do not seem to be affected or show any signs of neovessel formation.

The amount of 7KCh in these implants is very high but surprisingly within the physiological range found in atheromatous plaques. Most of the studies that have reported the 7KCh levels in atheromatous plaques have unfortunately used saponification [15], [16]. Saponification (methanolic KOH hydrolysis) or even mild alkaline treatments significantly destroys 7KCh [23] forming cholesta-3,5-diene-7-one which also further breaks down into other artifacts. This has resulted in significant underestimation of 7KCh content in many of the published studies. Saponification under argon in the presence of EDTA and BHT does not prevent the breakdown of 7KCh (unpublished results). A recent study analyzed the atheromatous plaques without saponification and has reported considerably higher levels of 7KCh [17]. In this study, human atheromatous lesions were reported to contain 25 mg of 7KCh per gram of wet weight or 2.5% [17]. In dry weigh equivalent, the levels in these plaques would likely be in double digit percent. Moreover, this study [17] did not quantify the 7KCh-fatty acid esters, which previous studies [3], [16] have shown to be abundant in oxidized LDL. Therefore, while our implants are obviously designed to induce an acute inflammatory response, our results demonstrate the dangers that oxidized lipid deposits with high 7KCh content could pose to cells.

Recently, Lutty and colleagues [24] developed a choroidal neovascularization model in rats and rabbits by subretinal injections of 13(s)-hydroperoxy-9z-11e-octadienoic acid. This compound is an oxidized form of linoleic acid which has previously been reported to be present in abnormally high levels in aged Bruch's membrane and choriocapillaris [25]. This is an elegant model that further supports our overall hypothesis that oxidized lipids present in oxidized lipoprotein deposits may be age-related risk factors in the pathogenesis of age-related macular degeneration.

In summary our *in vivo* data supports the previously published *in vitro* work regarding the cytotoxicity and inflammatory responses to 7KCh. This model also establishes an *in vivo* platform for further studies into the mechanisms of7KCh- and oxidized LDL-related angiogenesis and inflammation. In addition this model can also be used to study anti-angiogenic drugs. In preliminary studies we have successfully used this model to test various anti-angiogenic drugs by either mixing the drug with the 7KCh in the implant or by topical delivery via eye drops.
